# Validity of Bioelectrical Impedance Analysis to Estimation Fat-Free Mass in the Army Cadets

**DOI:** 10.3390/nu8030121

**Published:** 2016-03-11

**Authors:** Raquel D. Langer, Juliano H. Borges, Mauro A. Pascoa, Vagner X. Cirolini, Gil Guerra-Júnior, Ezequiel M. Gonçalves

**Affiliations:** Growth and Development Laboratory, Center for Investigation in Pediatrics (CIPED), School of Medical Sciences, University of Campinas (UNICAMP), Campinas-SP 13083-887, Brazil; borgesedfisica@gmail.com (J.H.B.); pascoawaf@bol.com.br (M.A.P.); vxcirolini@hotmail.com (V.X.C.); gilguer@fcm.unicamp.br (G.G.-J.); emaildozeique@gmail.com (E.M.G.)

**Keywords:** body composition, predictive equations, dual-energy X-ray absorptiometry

## Abstract

Background: Bioelectrical Impedance Analysis (BIA) is a fast, practical, non-invasive, and frequently used method for fat-free mass (FFM) estimation. The aims of this study were to validate predictive equations of BIA to FFM estimation in Army cadets and to develop and validate a specific BIA equation for this population. Methods: A total of 396 males, Brazilian Army cadets, aged 17–24 years were included. The study used eight published predictive BIA equations, a specific equation in FFM estimation, and dual-energy X-ray absorptiometry (DXA) as a reference method. Student’s *t*-test (for paired sample), linear regression analysis, and Bland–Altman method were used to test the validity of the BIA equations. Results: Predictive BIA equations showed significant differences in FFM compared to DXA (*p* < 0.05) and large limits of agreement by Bland–Altman. Predictive BIA equations explained 68% to 88% of FFM variance. Specific BIA equations showed no significant differences in FFM, compared to DXA values. Conclusion: Published BIA predictive equations showed poor accuracy in this sample. The specific BIA equations, developed in this study, demonstrated validity for this sample, although should be used with caution in samples with a large range of FFM.

## 1. Introduction

Adequate assessment of body composition is very important for the identification of possible health risks related to the excess or lack of different body components. It also helps in monitoring the processes of growth and aging and of some diseases, providing important evaluation data of nutritional interventions and physical exercise programs [1,2].

Many changes occur in body composition throughout life, mainly in the fat mass (FM) to fat-free mass (FFM) ratio [3]. Increased body fat is associated with the increased risk of various diseases, such as obesity, cardiovascular disease, type 2 diabetes, hypertension, and others [4]. FFM is comprised mostly by muscle mass and is related to the prevention of the fall risk in the elderly, as well as having significant influence on physical performance [5]. The evaluation of this body component can contribute to the development, monitoring, and improvement of physical training programs [6], as well as in the reduction of body fat [7].

A military career demands adequate levels of physical fitness and body composition. Due to this, young military members (cadets), undergo rigorous physical training programs when they join the military service [8,9], with the objective to efficiently perform the tasks of the proposed military routines. As a result, the FFM of these individuals increases, improving their physical performance [10] and reducing the FM percentage [11].

Body composition assessment is usually obtained by laboratory methods, such as dual-energy X-ray absorptiometry (DXA), plethysmography, hydrostatic weighing, and multi-compartmental models (four, five, or six compartments) [12,13]. Due to their complexity, they require specially-trained users for application [7]. The assessment is made only in laboratory or clinical facilities due to the impossibility to perform repeated measures in a small amount of time, and the high cost, making it difficult to use with large samples. On the other hand, bioelectrical impedance analysis (BIA) is a non-invasive, relatively simple, and widely used technique to estimate body composition in larger groups with individuals of different characteristics, such as gender, age, ethnicity, presence of diseases [7,13,14,15,16,17,18,19,20], and also in military [21,22].

BIA is a recommended method for field studies because it facilitates evaluation of a large number of individuals in a short period of time [23]. It can be used inside or outside the laboratory or clinical facilities [24]. Estimation of body composition by BIA is based on predictive equations developed in different populations with specific characteristics (e.g., gender, age, ethnicity, and anthropometry). It shows high predictive errors when applied to a population with diverse characteristics from those of the population in which the predictor was developed [25].

Therefore, the aims of this study were to verify the accuracy of predictive equations of BIA, already published for the estimation of FFM, in male Army cadets, aged 17 to 24 years, and to develop and cross-validate a specific BIA equation for this population, using DXA with a reference method. We believe that, despite the specific characteristics observed in our sample (young adults with low fat and high physical activity), this predictive model may be used for other subjects of the same age, such as military personnel, and in physically-active individuals (not athletes), as there is are lack of equations for this population.

## 2. Methods

### 2.1. Subjects

Data was collected during the beginning of the school year (March) of 2013 and 2014, when the annually “Preparatory School of Army Cadets” (EsPCEx), Campinas-SP, receive 500 students coming from all regions of Brazil. All cadets that entered in both years (2013 and 2014) were invited to participate in this study (approximately 1000 subjects); 946 showed an interest to participate and were included. From this total, participants were excluded if: (a) they did not have conditions or availability to participate in all procedures of the study protocol (*n* = 507) or (b) did not return a signed Terms of Informed Consent Form (*n* = 43). Thus, the final sample consisted of 396 volunteers.

### 2.2. Study Design and Ethics

It is a cross-sectional study, in which individual collections of anthropometry, BIA, and DXA data were done in the same day. This research was approved by the Ethics Committee of the School of Medical Sciences, University of Campinas (UNICAMP). All procedures were conducted according to the declaration of Helsinki [26] for studies with human subjects.

### 2.3. Measurements

Body weight (kg) was determined by a digital scale to nearest 0.1 kg and height (cm) by a vertical stadiometer to nearest 0.1 cm, following the recommended protocols [27]. Body mass index (BMI kg/m^2^) was calculated.

Body composition was determined by an iDXA (GE Healthcare Lunar, Madison, WI, USA) and version 13.6 enCore™ 2011 software (GE Healthcare Lunar). Total body measurements were performed to determine FM, bone mineral content (BMC), and lean soft tissue (LST). The FFM was obtained by the sum of the BMC and LST (FFM = BMC + LST) values. The reproducibility of the variables estimated by DXA was determined by the coefficient variation (CV%) and technical error of measurement (TEM), based on the test-retest realized with 23 subjects out of the population of this study. The formula for TEM is $\;=\;\sqrt{\frac{D^{2}}{n}}$, where *D* is the difference between the two measurements, and *n* is the sample size. The CV% were 0.74%, 0.28%, and 0.26% to FM, BMC, and LST, respectively, and TEM were 0.25 kg, 0.02 kg, and 0.25 kg to FM, BMC, and LST, respectively.

BIA measurements were performed according to the protocol recommended by Kyle [24]. We used a tetrapolar device, single frequency (50 kHz), and model Quantum II (RJL Systems, Detroit, MI, USA). BIA provides resistance (*R*) and reactance (*Xc*) values in Ohms (Ω). Reproducibility was calculated for a subgroup of this study population (23 subjects); CV of 0.35% and 0.33%, for *R* and *Xc*, respectively; TEM of 3.54 Ω and 0.49 Ω, for *R* and *Xc*, respectively; and impedance (*Z*) was calculated by the formula: $Z\;=\;\sqrt{R\;+\;Xc}$.

### 2.4. Selection of Predictive Equations of BIA

BIA predictive equations were selected adopting these criteria: (a) subjects with age compatible with the sample of the present study; (b) sample involving male subjects; and (c) BIA equipment from the same manufacturer and same frequency (50 kHz). We selected eight equation, previously published in the literature. The characteristics are shown in Table 1.

### 2.5. Statistical Analysis

Data were analyzed using IBM SPSS Statistics version 16.0 (IBM, Chicago, IL, USA). With the exception of FFM of DXA and Equation (3) [15] values, other equations did not show normal distribution; for this reason the logarithmic transformation (log^10^) was used. The paired Student’s *t*-test for paired samples was used to verify the differences between the estimated values by BIA predictive equations and the values determined by DXA. The adjusted coefficient of determination (*R*^2^) and the standard error of estimated SEE were obtained using simple linear regression. The pure error (PE) was assessing using the following equation [28]:

$$PE=\sqrt{\begin{array}{c} \frac{\sum {\left(Ÿ-Y\right)}^{2}}{n} \end{array}}$$

where variable Ÿ is estimated FFM of predictive equations of BIA, *Y* is FFM of DXA, and *n* is the total number of subjects in the sample. Lin’s approach [29] for the concordance correlation coefficient (CCC) was calculated using MedCalc Statistical Software v.11.1.0, 2009 (Mariakerke, Belgium), to verify the accuracy (*C_b_*) and precision (*ρ*) between the estimated FFM values by BIA and determined by DXA. The Bland–Altman [30] method was used to verify the agreement between the estimated FFM values by BIA and determined by DXA, and bivariate Pearson’s correlation (*r*) were conducted to determine whether the difference between each predictive equation and the reference method was related to the mean of the two measurements (trends). For development and cross-validation of the specific BIA equation, we followed the proposed recommendations by Sun and Chumlea [28], in which the total sample (*n* = 396) was randomly distributed by a statistical program into two groups: development group (DG) (*n* = 264) and cross-validation group (VG) (*n* = 132). Multiple linear regression analysis (stepwise method) was used for the development of the new model. The adequacy of the final prediction model was assessed by testing the normality of the residuals by a Shapiro–Wilk test. All of this parameters (paired Student’s *t*-test, *R*^2^, SEE, PE, and Bland–Altman) were considered to evaluate the accuracy of the eight predictive equations of BIA and the specific equation developed in this study. *p* < 0.05 was considered statistically significant.

## 3. Results

Table 2 presents the general characteristics of the total sample and groups (development and cross-validation) for the specific BIA equations. No significant differences were found between development and cross-validation groups.

The independent variables age, height, BMI, *R*, *Xc*, and impedance on the development group showed significant correlations (*p* < 0.01) with FFM determined by DXA, however, of low or moderate intensity, with r values ranging from 0.16 to 0.66. The variables with higher correlation were: body weight (kg) (*r* = 0.92) and stature^2^/*R* (cm^2^/Ω) (*r* = 0.84) (*p* < 0.001). The second variable was transformed into a base-10 logarithm (log^10^). The resulting specific BIA equation by stepwise regression analysis was:

FFM = 0.508 × *Wt* + 39.234 × (stature^2^/*R*)^Log10^ − 48.263

The FFM values determined by DXA showed significant difference (*p* < 0.05) compared to FFM values of the eight BIA predictive equations. Two equations (Equations (4) and (5)) showed lower values and six equations (Equations (1)–(3) and (6)–(8)) showed higher values of FFM. The FFM average estimated by the specific equation developed in this study showed no significant difference compared to the value determined by DXA, to both development and cross-validation groups, as in the total sample. It was observed that the value of CCC above 0.80 existed between the predictive equations and the reference method, while in the development and cross-validation group, as well as in the total sample, the CCC values were above 0.92 (Table 3).

**Figure 1:**
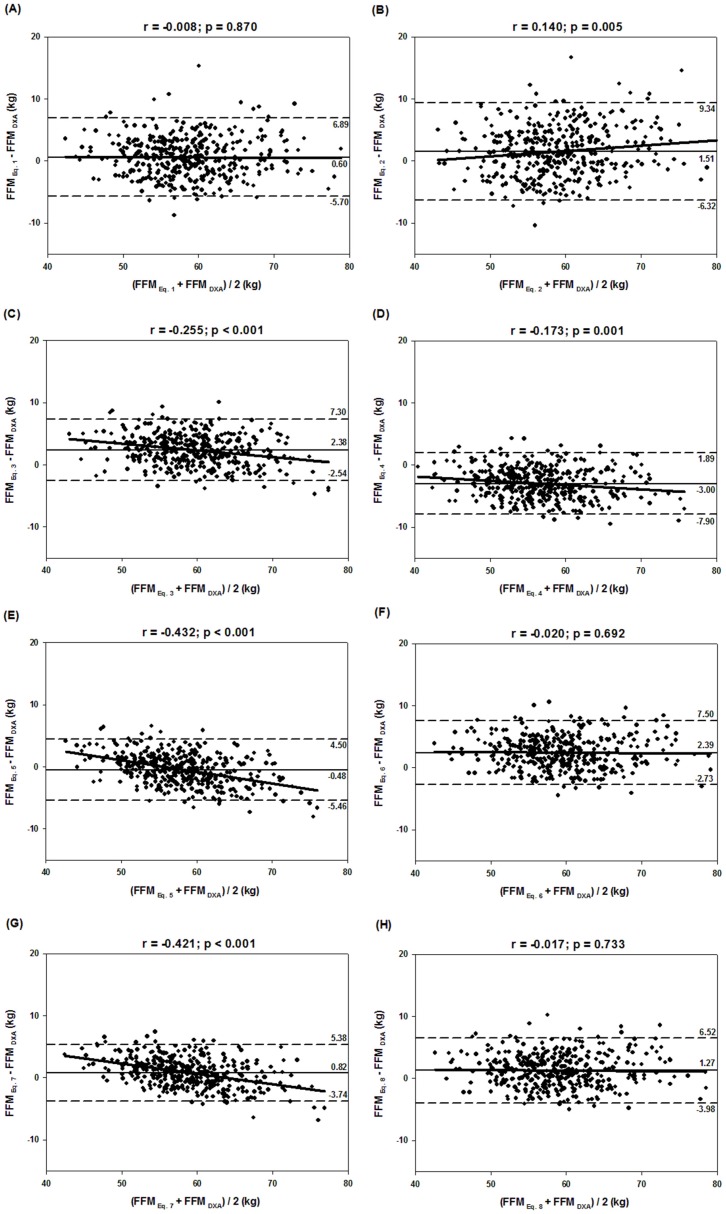
Bland–Altman plots of the agreement between values of FFM determined by the reference method (DXA) and estimated by BIA equations in the sample of cadets (*n* = 396): (**A**) Equation (1) [13]; (**B**) Equation (2) [14]; (**C**) Equation (3) [15]; (**D**) Equation (4) [16]; (**E)** Equation (5) [17]; (**F**) Equation (6) [18]; (**G**) Equation (7) [19]; (**H**) Equation (8) [20]. Solid black line: mean of the differences; dashed line: limits of agreement of 95%; continuous gray line: correlation (*r*) between the average and the differences of the methods.

Figure 1 shows the agreement of methods (DXA and BIA) using the Bland–Altman analysis. All BIA equations showed large limits of agreement. In addition, Equations (2)–(5) and (7) showed significant trends (*p* < 0.05) between the differences and mean of methods, as observed from the *r* value.

**Figure 2:**
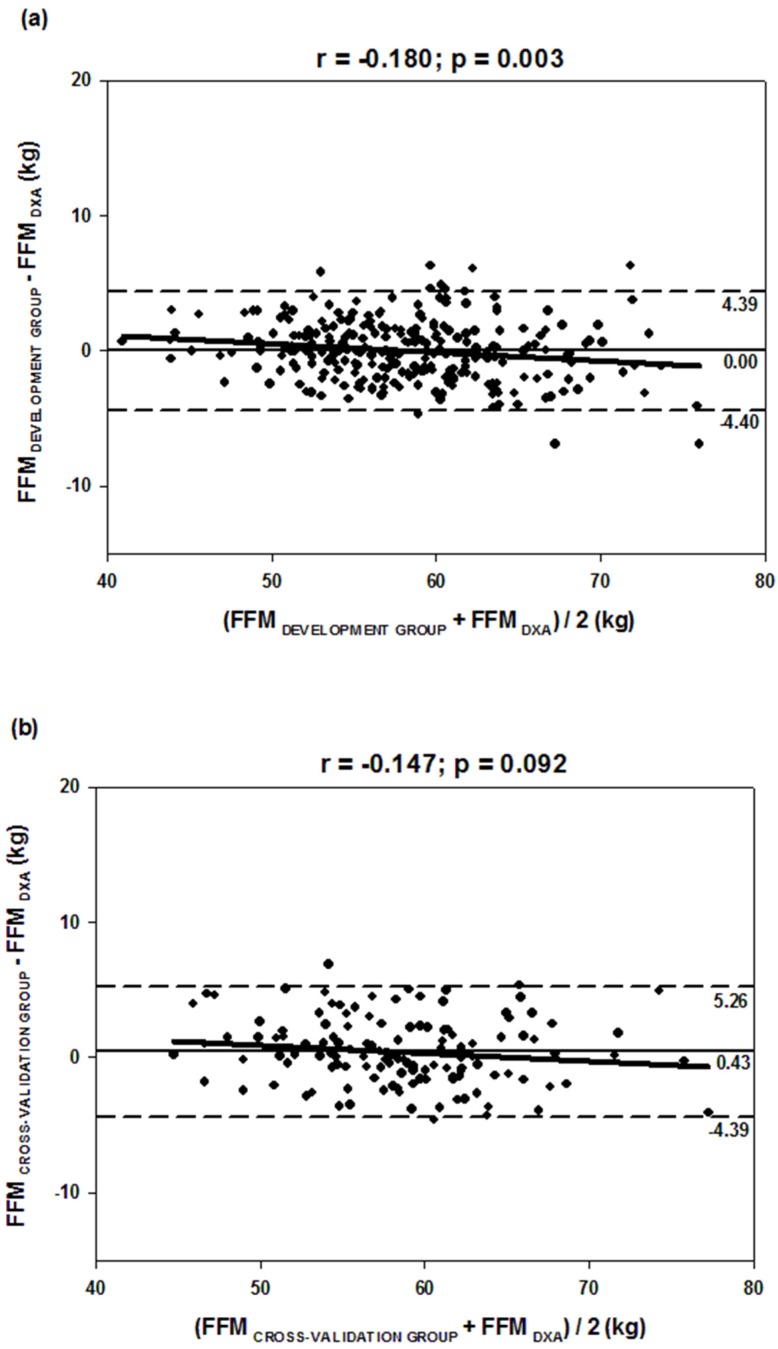
Bland–Altman plots of the agreement between values of FFM determined by the reference method (DXA) and estimated by BIA-specific equations: development group (1); and cross-validation group (2). Solid black line: mean of the differences; dashed line: limits of agreement of 95%; continuous gray line: correlation (*r*) between the average and the differences of the methods.

Figure 2 shows the agreement of methods (DXA and BIA) using the Bland–Altman analysis. The two groups (development and cross-validation) showed wide limits of agreement, but only development group (a) showed a significant trends (*p* < 0.05) between the differences and mean of methods, as observed from the *r* value.

## 4. Discussion

The aim of this study was to test the accuracy of eight predictive equations, available in the literature, based on the BIA to estimate FFM in young males, Brazilian Army cadets, using DXA as a reference method. The values estimated by these eight predictive BIA equations correlated strongly with the DXA values. We observed significant differences between the FFM values of all equations when compared to the reference method. Furthermore, they did not show good agreement with DXA. Several authors reported strong correlation between FFM estimated by BIA and DXA [19,31,32], and as observed in our study (Figure 1), they also found great individual variability confirmed by large limits of agreement [32].

Body composition assessment by BIA is based on the relative stability of hydration of the FFM (ratio of body water per FFM). However, BIA was not developed for FFM assessment, as it measures water that is extrapolated for an amount of FFM. The electrical conduction of the body water depends on the amount of electrolytes [23]; the amount of body water varies with each age range [33]. In healthy adults this ratio is considered stable at a value of 0.73, and may vary between 0.69 and 0.77 [34]. Although this small variability, it can increase the error of predicting body composition, especially in children and young adults [35], elderly [33], and in subjects with diseases, according to the state of hydration [36]. Thus, it is very important to carefully select the equation to be used, making sure that they have been developed from similar samples considering age, gender, ethnicity, and health status [36,37].

Our sample was composed only of young and healthy male adults; the admission process to these individuals be accepted in EsPCEx consisted of three phases: the first was comprised of an intellectual test; the second by a medical inspection in which the candidate must submit an “Authorization for realization of health inspection”, signed by the responsible party, with the report of various medical exams (exercise testing, complete blood count, electroencephalogram, radiography of the lungs, *etc.*); and the third physical test (muscular strength and endurance, and of aerobic fitness) to check the level of general physical fitness. Based on these facts, we considered our sample to be homogeneous in relation to age, health, and level of physical activity, factors that can interfere with the FFM estimated using the BIA in heterogeneous samples [37]. Although homogeneous, our sample exhibited great individual variability, confirmed by the large limits of agreement obtained in the Bland–Altman [30] analysis and due to differences to be dependent on the amount of FFM of these individuals. We observed a lower agreement between the methods that assess the FFM of predictive equations and DXA (ranged between CCC = 0.80 and CCC = 0.90); for the specific equation developed in this study, we observed a strong agreement between the methods (CCC = 0.93).

As well as in the present study, several previous studies have developed FFM prediction models using the BIA [13,15,16,17,18,19,20], with body weight and index of stature^2^/resistance variables. These variables presented the best predictive ability for the development of the specific equation of our sample. One of the BIA’s assumptions is that the human body has a format similar to a perfect cylindrical conductor [38]; however, the human body shape resembles a shape comprise more of a series of five connected cylinders (two arms, two legs and trunk, head excluded) [24]. Body segments are not uniform (different shapes and sizes), so the resistance to current flow in the body segments are different [24]. One of the factors that may have influenced the wide variation in the results estimated by BIA predictive equations is that, in our sample, individuals came from all regions of the country (north, northeast, midwest, southeast, and south) and presented different characteristics relative to the body segments. This heterogeneity observed in previous studies seems to contribute to the lack of applicability of BIA equations from one population to the other [24,37].

The greatest problem found in the prediction FFM by BIA equations in our sample was the wide variability among individuals observed in the Bland–Altman plots, with 95% limits of agreement ranging from −5.8 kg to 5.8 kg. These results indicates that some individuals can have their FFM values overestimated by more than nine kilograms (Equation (2)) and underestimated by more than eight kilograms (Equation (4)), which is an error of 16% and 14% for more and less, respectively, when comparing the average of the FFM values determined by DXA. This wide variation between individuals was also observed in other studies that have developed new equations of BIA using DXA as a reference in children and adolescents [31], obese children and adolescents [39], and 12–19 years old boys of different ethnicities [37]; these models should be considered when using BIA equations.

With the exception of the Equations (1), (6), and (8), the differences observed were significantly correlated with the average of the two methods, although these correlations are low (Equations (2)–(4) and this study’s specific equation) or moderate (Equations (5) and (7)), indicating that the observed bias is dependent on the quantity of FFM and results different to individuals with diverse levels of FFM, overestimating with higher amounts of FFM (Equations (2)), or underestimating these same individuals (Equations (3)–(5), (7) and this study’s specific equation). Predictive BIA equations should be used with caution in groups with wide variation in FFM [36]. In this sense, despite the results that the specific BIA equation did not show significant bias with DXA, they showed a significant trend in observed differences, which in practice means that these differences are dependent on the amount of FFM of the individuals.

Various factors can affect the results of BIA: no standardization of body position, previous exercise, and food intake [36,38]; in this study all of these factors were controlled. Another source of error can be the reference method used in the development of the BIA equations [36]. The recommendation is that the validation BIA equations are performed against reference using methods incorporating the model 4C [40,41], densitometry (hydrostatic weighing and plethysmography), DXA, and isotope dilution of body [40]. Each of these reference methods is not without error and have their limitations [41]. In the present study, the majority of the tested BIA equations used densitometry by hydrostatic weighing (Equation (1) to (6)); only Equation (7) used DXA and Equation (8) used the 4C model. The variation may be related to greater or lesser validity of the reference method. The BIA equations have been developed for a specific population and that may or not be comparable to other reference methods [36].

Several studies have demonstrated the potential of DXA for assessing total and regional body composition due to the relative speed of scanning, low radiation exposure, and good accuracy and reproducibility of the measurements [42,43]. However, although the DXA is considered as a reference method for the estimation of bone mass [44], it may have limitations in the determination of FFM and FM [45]; studies that compared the ratings for multi-compartmental methods observed that both the FM and the %FM were overestimated in the assessment with DXA [46].

As well as this study, several other studies, especially in recent decades, have used DXA as the reference method to develop new equations of BIA in healthy children and adolescents [47], and obese [39] subjects of different ethnic groups [31,32,37] and, some with diseases [31]. The 4C model would be the reference method of choice, but due to its complexity [12] it has rarely been applied to develop equations based on the BIA [20]. Methods based on the division of the body in two compartments (hydrostatic weighing and isotope dilution) do not consider the changes in the hydration of the FFM, influenced by gender, age, and maturity [16,17,24]. In this context, the DXA provides estimation of body composition relatively independently of body hydration [40].

A limiting factor of this study is the miscegenation in Brazil, which is a very heterogeneous country as a result of more than five centuries of miscegenation of people from different ethnic groups coming from various continents (Europe, Africa, and Asia) in addition to near 2.5 million American Indians who already lived in the country. With this, from the very beginning of the 1990s, the country has officially adopted the proposal that these data must be collected based on self-declaration, *i.e.*, each individual choose between five categories of color skin—white, black, “mulato”, yellow, and indigenous—what he or she feels appropriate [48]; but as this classification is different from the ones used in other countries, it is very difficult to establish comparisons relating the influence of ethnicity on our results. This also explains, in part, the need for specific regression models to the characteristics of the population evaluated, due to the differences in body proportions related to age, gender, and ethnicity [36]. Another limitation of this study is that the subjects were studied on a single occasion; it would be interesting the assessment at two time-points, to see if these results have the same behavior on changes in FFM over time.

## 5. Conclusions

BIA it is a non-invasive, portable, and relatively inexpensive method, and can be used in individuals with different characteristics, provided that specific equations are validated and appropriate with respect to age, sex, and ethnicity. The eight BIA prediction equations, already published in the literature, were not valid to this Brazilian Army cadets sample. Due to the lack of specific BIA equations in the literature for this population, we suggested new longitudinal studies assessing the validity of the BIA to verify the changes in the quantity of FFM. Although the specific equation developed in this study did not show significant differences with the reference method, wide limits of agreement and bias, dependently on the quantity of FFM, were observed, and it should be used with caution, especially if it is used on samples with different characteristics of the subjects in this study.

## Figures and Tables

**Table 1:** Characteristics of predictive equations of BIA selected for the estimation of FFM.

Initials	Reference	Sex (*n*) M/F	Age (Years)	Criterion	Prediction Equation of Fat-Free Mass	*R*^2^	SEE
Equation (1)	Lukaski, *et al.* [13]	321 ^b^	18–73	UW	0.734 × (*S*^2^/*R*) + 0.116 × *Wt* + 0.096 × *Xc* + 0.878 × Sex ^c^ − 4.03	0.99	2.2
Equation (2)	Chumlea, *et al.* [14]	77/96	18–62	UW	0.87 × (*S*^2^/*Z*) + 3.50	0.81	3.0
Equation (3)	Segal, *et al.* [15]	1069/498	17–62	UW	0.00132 × *S*^2^ − 0.04394 × *R* + 0.3052 × *Wt* − 0.1676 × Age + 22.66827	0.9 ^d^	3.6
Equation (4) ^a^	Deurenberg, *et al.* [16]	130/116	7–25	UW	0.438 × (*S*^2^/*Z*) + 0.308 × *Wt* + 1.6 × Sex + 7.04 × *S*) − 8.50	0.99	2.4
Equation (5) ^a^	Deurenberg, *et al.* [17]	361/466	16–83	UW	0.34 × (*S*^2^/*Z*) − 0.127 × Age + 0.273 × *Wt* + 4.56 × Sex ^c^ + 15.34 × *S* − 12.44	0.93	2.6
Equation (6)	Lohman [18]	153/153	18–30	UW	0.485 × (*S*^2^/*R*) + 0.338 × *Wt* + 5.32	NR	2.9
Equation (7)	Kotler, *et al.* [19]	206/126	18–40	DXA	0.50 × (*S*^1.48^/*Z*^0.55^) × (1.0/1.21) + 0.42 × *Wt* + 0.49	0.9 ^d^	5.0 ^e^
Equation (8)	Sun, *et al.* [20]	734/1095	12–94	4C	0.65 × (*S*^2^/*R*) + 0.26 × *Wt* + 0.02 × *R* − 10.68	0.90	3.9

Abbreviations: *n*, number of subjects; M, male; F, female; UW, underwater weighing; DXA, dual-energy X-ray absorptiometry; 4C, model four compartments; S, stature (cm); *R*, resistance (Ω); *Xc*, reactance (Ω); Z, impedance (Ω); Wt, weight (kg); *R*^2^, coefficient of determination; NR, not reported; SEE, standard error of estimated in kilograms; ^a^ equations that used the value of the height in meters (m); ^b^ male and female subjects; ^c^ 0 if female and 1 if male; ^d^ correlation coefficient value (*r*); ^e^ value in percentage (%).

**Table 2:** Characteristics of the total sample and the groups (development and cross-validation) of specific BIA equations.

Variables	Total (*n* = 396)	Development (*n* = 264)		Cross-Validation (*n* = 132)	
	Mean ± SD	Mean ± SD	Min–Max	Mean ± SD	Min–Max
Age (years)	19.2 ± 1.8	19.3 ± 1.2	17.0–24.0	19.1 ± 1.1	17.0–24.0
Weight (kg)	70.0 ± 8.5	69.9 ± 8.5	45.9–94.8	70.3 ± 8.7	50.9–99.4
Stature (cm)	175.8 ± 6.4	176.0 ± 6.7	159.6–192.7	175.3 ± 5.9	160.2–190.8
BMI (kg/m^2^)	22.6 ± 2.3	22.5 ± 2.2	16.0–29.7	22.8 ± 2.4	17.1–32.3
FM (%)	17.2 ± 3.9	17.1 ± 3.7	9.0–27.6	17.3 ± 4.2	10.2–27.8
BMC (kg)	3.0 ± 0.4	3.0 ± 0.4	2.0–4.2	3.0 ± 0.4	2.2–4.2
LST (kg)	55.2 ± 6.2	55.2 ± 6.2	38.5–75.4	55.2 ± 6.0	41.6–75.5
FFM (kg)	58.2 ± 6.5	58.2 ± 6.5	40.6–79.5	58.3 ± 6.4	44.0–79.3
Resistance (Ω)	479.5 ± 48.8	483.8 ± 48.4	345.0–669.0	470.8 ± 48.7	349.0–665.0
Reactance (Ω)	62.4 ± 7.0	63.0 ± 6.7	40.0–86.0	61.3 ± 7.7	27.0–80.0
Impedance (Ω)	483.6 ± 48.9	487.9 ± 48.5	349.5–673.4	474.9 ± 48.8	353.8–668.9

Abbreviations: BMI, body mass index; FM, fat mass; BMC, bone mineral content; LST, lean soft tissue. FFM, Fat-free mass.

**Table 3:** Values of fat-free mass estimated by BIA equations and determined by DXA.

	Fat-Free Mass		Difference		CCC Analysis			*R*^2^	SEE (kg)	PE (kg)
	Mean ± SD	Min–Max	Mean ± SD	%	CCC	*ρ*	*C_b_*			
**Equation (1)**	58.8 ± 6.5 ^a^	44.1–79.9	0.6 ± 3.2	1.0	0.87	0.8762	0.9957	0.77	3.1	3.3
**Equation (2)**	59.7 ± 7.0 ^a^	42.9–82.7	1.5 ± 4.0	2.6	0.80	0.8271	0.9724	0.68	3.6	4.3
**Equation (3)**	60.6 ± 5.8 ^a^	45.0–75.6	2.4 ± 2.5	4.1	0.85	0.9219	0.9259	0.85	2.5	3.5
**Equation (4)**	55.2 ± 6.1 ^a^	40.3–72.5	−3.0 ± 2.5	5.2	0.83	0.9225	0.8945	0.85	2.5	3.9
**Equation (5)**	57.7 ± 5.4 ^a^	44.1–72.7	−0.5 ± 2.5	0.8	0.91	0.9242	0.9805	0.85	2.5	2.6
**Equation (6)**	60.6 ± 6.4 ^a^	44.5–79.8	2.4 ± 2.6	4.1	0.86	0.9181	0.9355	0.84	2.6	3.6
**Equation (7)**	59.0 ± 5.5 ^a^	44.3–74.7	0.8 ± 2.3	1.4	0.92	0.9371	0.9781	0.88	2.3	2.5
**Equation (8)**	59.5 ± 6.4 ^a^	44.1–78.9	1.3 ± 2.7	2.2	0.90	0.9138	0.9808	0.84	2.6	3.0
**Equation Specific**										
**DG**	58.2 ± 6.1	41.3–75.0	0.0 ± 2.2	0.0	0.94	0.9392	0.9980	0.88	2.2	2.2
**CVG**	58.7 ± 6.0	44.8–76.7	0.4 ± 2.5	0.7	0.92	0.9228	0.9959	0.85	2.5	2.5
**Total**	58.4 ± 6.1	41.3–76.7	0.1 ± 2.3	0.2	0.93	0.9334	0.9979	0.87	2.3	2.3

Abbreviations: CCC, concordance correlation coefficient; *ρ*, precision; *C_b_*, accuracy; SEE, standard error of estimated; PE, pure error; DG, development group; CVG, cross-validation group. ^a^ significant difference of DXA (58.2 ± 6.5), paired Student’s *t* test (*p* < 0.05).
